# Effect of ultrasound-guided lumbar plexus block on emergence agitation in children undergoing hip surgery: study protocol for a randomized controlled trial

**DOI:** 10.1186/s13063-018-3140-3

**Published:** 2019-01-07

**Authors:** Hui Zhang, Qing Fan, Junfeng Zhang, Bin Wu, Xiaofeng Wang, Yu Zhang, Aizhong Wang

**Affiliations:** 1 Department of Anesthesiology, Shanghai Sixth People’s Hospital Affiliated to Shanghai Jiao Tong University, No. 600, Yishan Road, Shanghai, 200233 China; 2 Department of Pediatric Orthopedics, Xinhua Hospital Affiliated to Shanghai Jiao Tong University School of Medicine, Shanghai, 200092 China

**Keywords:** Emergence agitation, Ultrasound, Lumbar plexus block, Pediatrics, Hip surgery, Randomized controlled trial

## Abstract

**Background:**

Emergence agitation (EA) is a common postoperative issue in children that causes self-injury, increases stress on healthcare team members, and even leads to postoperative maladaptive behavioral changes in children. Clear answers regarding a ‘gold standard’ for prevention of EA are not available. Pain is regarded as an important causative factor of EA, and ultrasound-guided lumbar plexus block is a safe and efficient anesthetic method that can provide satisfactory pain relief in pediatric hip surgery. The purpose of our study is to determine whether ultrasound-guided lumbar plexus block can reduce the incidence of EA in children undergoing hip surgery.

**Methods/design:**

We designed a prospective, randomized, controlled, blinded trial to determine the effect of ultrasound-guided lumbar plexus block on EA. A total of 100 American Society of Anesthesiologists class I–II children (1–6 years old) scheduled for elective hip surgery will be recruited for this study. Participants will be randomized at a 1:1 ratio to receive either ultrasound-guided lumbar plexus block or fentanyl after the induction of general anesthesia. The primary outcome is the incidence of EA 30 min after emergence from anesthesia using the Pediatric Anesthesia Emergence Delirium (PAED) score. The secondary outcomes are the severity and duration of EA 30 min after emergence from anesthesia using the PAED score, postoperative pain evaluated by the Children’s Hospital of Eastern Ontario Pain Scale, and the incidence of postoperative adverse events. Randomization will be conducted using a computer-generated randomization schedule. Outcome assessors and data collectors will be blinded to the group allocations. Assessments will be performed before surgery, intraoperatively, and postoperatively at every time point.

**Discussion:**

Our hypothesis in this trial is that ultrasound-guided lumbar plexus block can decrease the incidence of EA in children undergoing elective hip surgery. This trial will provide clinical answers to verify our hypothesis. If our hypothesis is confirmed, the results could provide a safe method to prevent EA.

**Trial registration:**

Chinese Clinical Trial Registry, ChiCTR-INR-17011525. Registered on 30 May 2017.

**Electronic supplementary material:**

The online version of this article (10.1186/s13063-018-3140-3) contains supplementary material, which is available to authorized users.

## Background

Emergence agitation (EA) is a common phenomenon in the early postanesthetic period in children aged 3–9 years, which was first described by Eckenhoff et al. [1] in the early 1960s. The incidence of EA ranges from 10 to 80% [2, 3]. Although EA is short-lived, as it lasts an average of 30 min and is self-limiting [4], it may cause children to be prone to self-injury, interfere with a child’s recovery, increase healthcare costs, present a challenge to pediatric anesthesiologists, and decrease parents’ satisfaction with anesthesia [5–10]. Furthermore, EA is associated with postoperative maladaptive behavioral changes [11] and was recently considered a ‘vital sign’ [12]. Methods to prevent and treat EA have gained increased interest in pediatric anesthesia, but no gold standard method is currently available.

A variety of studies have investigated numerous contributors to EA, but the exact etiology of EA remains unclear. Inhalational agents with low blood solubility and pain are regarded as two significant predisposing factors of EA. The frequency of EA in children has increased due to the growing use of sevoflurane in general anesthesia, and nerve block administration can decrease the amount of sevoflurane used [13]. Pain plays an important role in EA [14–16], and adequate pain control can reduce the incidence of EA [6]. Numerous studies have reported that lumbar plexus block can provide superior analgesia effects that last 14.5 h [17]. Ultrasound technology has increased in popularity in pediatrics for lumbar plexus block because ultrasound guidance can provide direct visualization of the lumbar plexus to ensure both the safety and efficacy of lumbar plexus block [18–20]. However, controversies concerning the effect of nerve block on EA exist [13, 21–23], and trials studying lumbar plexus block on EA have not been performed.

We designed this prospective, randomized, and blinded study in children undergoing elective hip surgery and hypothesized that ultrasound-guided lumbar plexus block may decrease the incidence of EA in children undergoing elective hip surgery. This trial will provide clinical answers to verify our hypothesis and recommendations for clinical practice.

## Methods

### Ethical aspects and informed consent

This trial was approved by the Ethics Committee of the Shanghai Sixth People’s Hospital affiliated to Shanghai Jiao Tong University. This study was registered at the Chinese Clinical Trial Registry (http://www.chictr.org.cn/) under number ChiCTR-INR-17011525 on May 30, 2017. All parents of eligible participants will be informed of the objective of trial, the randomization procedure, the workflow, and the potential risks and benefits of each intervention. We will obtain approval and written informed consent from parents of children scheduled for elective hip surgery.

### Study design

**Fig. 1:**
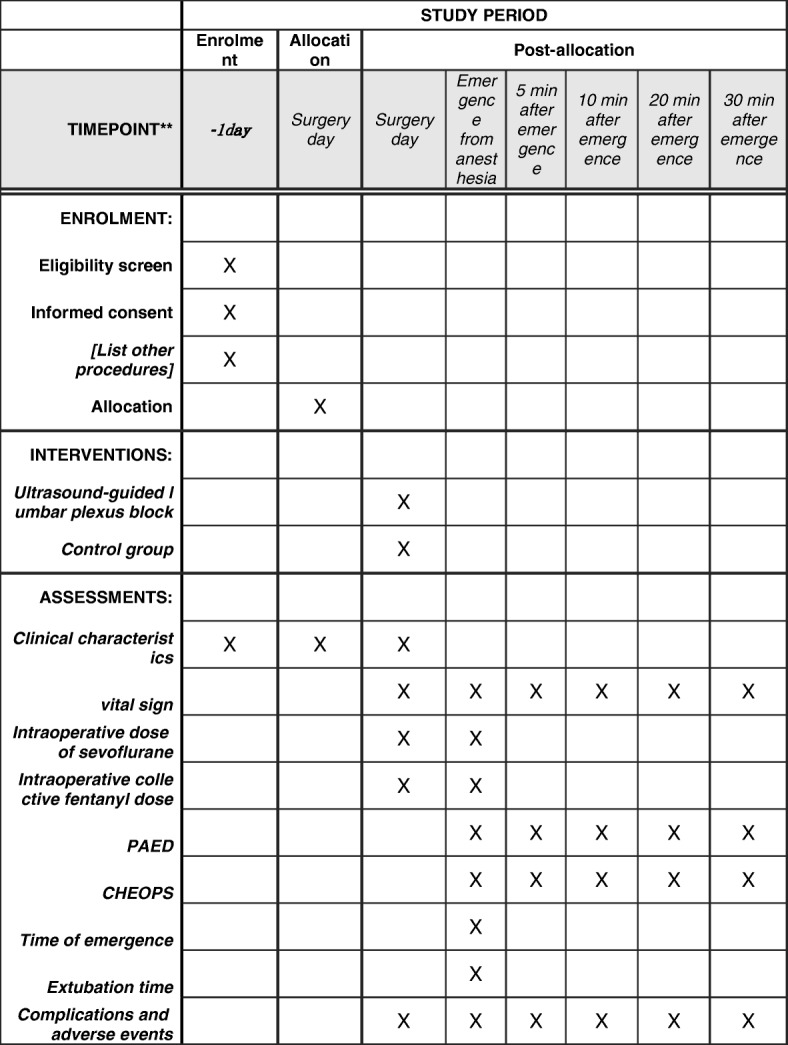
Standard Protocol Items: Recommendations for Interventional Trials schedule of enrollment, intervention, and assessments. CHEOPS Children’s Hospital of Eastern Ontario Pain Scale, PAED Pediatric Anesthesia Emergence Delirium

This study is designed as a single-center, prospective, randomized, controlled, blinded trial. The schedule of enrollment and assessments is presented in the Standard Protocol Items: Recommendations for Interventional Trials (SPIRIT) figure (Fig. 1 and Additional file 1).

### Objectives

#### Primary objective

The aim of our trial is to determine whether ultrasound-guided lumbar plexus block will reduce the incidence of EA in children undergoing elective hip surgery.

#### Secondary objectives

The secondary objectives are as follows:

- to compare the severity and duration of EA between the ultrasound-guided lumbar block group and the control group;
- to compare postoperative pain between the two groups; and
- to compare the occurrence of postoperative complications between the two groups.

### Study setting

This study will be conducted in Shanghai Sixth People’s Hospital affiliated to Shanghai Jiao Tong University, where approximately 200 pediatric hip surgeries are performed each year.

### Study population

Children scheduled for elective hip surgery will be recruited according to the following inclusion and exclusion criteria.

### Inclusion criteria

Prior to enrollment, patients must comply with all of the following:

- Undergoing elective hip surgery.
- Age 1–6 years.
- American Society of Anesthesiologists (ASA) class I–II.
- Informed parental consent provided.
- Suitable for general anesthesia and lumbar plexus block.

### Exclusion criteria

Patients are excluded if presenting any of the following:

- Lack of consent.
- Contraindication for lumbar plexus block.
- Developmental delay.
- Neurological or psychiatric disease.
- Local infection at the needle entry point.
- Coagulopathy.
- Nerve injury.
- Allergy to anesthetics and study medications.

### Outcomes and measurements

#### Primary outcome

The primary outcome of this study is the incidence of EA 30 min after emergence from anesthesia, which will be evaluated using the Pediatric Anesthesia Emergence Delirium (PAED) scale.

#### Secondary outcomes

The secondary outcomes are as follows:

- The severity and duration of EA 30 min after emergence from anesthesia evaluated using the PAED scale.
- The intensity of postoperative pain evaluated using the Children’s Hospital of Eastern Ontario Pain Scale (CHEOPS) scale.
- The incidence of postoperative adverse events.

### Randomization

Randomization will be based on computer-generated allocation, and random numbers will be concealed in opaque envelopes. After intubation, the envelope corresponding to a number in the randomization table will be opened, and the participant will be randomly assigned to either the ultrasound-guided lumbar block group or the control group at a 1:1 allocation ratio according to random number.

### Blinding

Due to the nature of our trial, it is not possible for the anesthesiologist who performs intraoperative anesthetic care to be blinded to the allocation. To minimize the possible bias, we will divide the research staff into two teams. Unblinded team: the anesthesiologist who performs intraoperative anesthetic care will know the assignment. The anesthesiologist is skilled in ultrasound-guided nerve block. The nerve block details will be recorded by the anesthesiologist and placed in a sealed envelope that will only be opened if medically necessary and at the completion of study. The anesthesiologist will not be involved in the outcome assessment. Blinded team: patients, parents, surgeons, assessment investigators, the medical staff who provide postoperative care in the postanesthesia care unit (PACU), data collectors, and statisticians will all be blinded to the group allocation.

### Study time

This study began in May 2017 and has been completed in October 2018. The study was conducted at Shanghai Sixth People's Hospital affiliated with Shanghai Jiao Tong University.

### Interventions

All patients will refrain from solid foods for 6 h and clear fluids for 2 h preoperatively. After intravenous access is established for all patients, standard monitoring—including electrocardiography (ECG), noninvasive blood pressure, and pulse oximetry (SpO_2_)—will be performed and recorded. The values of the heart rate, noninvasive blood pressure, and respiratory rate before anesthesia induction will be recorded as baseline values. During the intraoperative period, inspiratory and expiratory gas analysis and end-tidal carbon dioxide will also be monitored. All values will be recorded every 5 min. General anesthesia will be induced with 0.1 mg/kg midazolam, 2 μg/kg fentanyl, 3 mg/kg propofol, and 0.1 mg/kg vecuronium intravenously. Patients will be intubated endotracheally, and mechanical ventilation will be controlled to maintain end-tidal carbon dioxide at 35–40 mmHg. During the operation, anesthesia will be maintained with 60% nitrous oxide, 40% oxygen, and 2% end-tidal sevoflurane. After anesthesia induction, all children will be assigned to either the ultrasound-guided lumbar plexus block group or the control group according to the randomization table.

### Consort flowchart subject enrollment

**Fig. 2:**
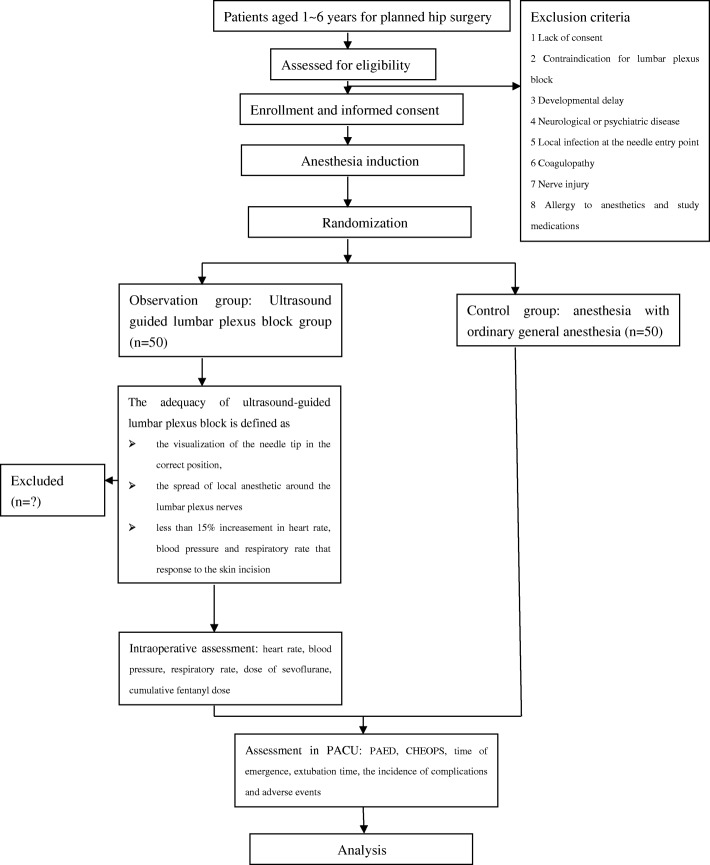
CONSORT flowchart of subject enrollment. CHEOPS Children’s Hospital of Eastern Ontario Pain Scale, PACU postanesthesia care unit, PAED Pediatric Anesthesia Emergence Delirium

The trial flow diagram is presented in Fig. 2.

### Ultrasound-guided lumbar plexus block group

Patients assigned to ultrasound-guided lumbar plexus block group will be placed in a lateral decubitus position with the hips flexed and the operative site facing upward. The S-Nerve™ Ultrasound System (Sonosite Inc., Bothell, WA, USA) will be used for the ultrasound scan. After aseptic preparation of the lumbar region and transducer, nerve block will be performed with a linear array (6–13 MHz) transducer using the longitudinal approach. All blocks will be performed by the same anesthesiologist experienced and qualified in ultrasound-guided lumbar plexus block. All investigators are trained at the beginning of the study to ensure a standardized intervention and minimize the potential bias.

A line will be drawn to connect the iliac crests and identify the fourth lumbar spine. The ultrasound probe will be placed lateral and parallel to the long axis of the spine at the level of L3–L4. The entry site will be 1.5–2 cm lateral to the midline along the intercristal line. The needle will be advanced cautiously perpendicular to the skin with ultrasound guidance until the tip of the needle reaches 1–1.5 cm below the space between the transverse processes of L3 and L4. To avoid a possible vascular puncture, repeated negative aspirations will be performed during all needle advancements. The lumbar transverse processes exhibit a hyperechoic reflection and acoustic shadows on ultrasound scans, which can produce a ‘trident sign’. The roots of the lumbar plexus are located in the posterior portion of the psoas muscle 1–1.5 cm below the space between the transverse processes. When the needle is in place, 1 ml/kg of 0.2% ropivacaine (Naropin 10 mg/ml; AstraZeneca, Wilmington, DE, USA) will be injected. The maximum dose of local anesthetic is limited to 20 ml, and the injection speed of ropivacaine will be approximately 1 ml/s.

The adequacy of the ultrasound-guided lumbar plexus block will be defined by the visualization of the needle tip in the correct position; the spread of local anesthetic around the lumbar plexus nerves; increases in the heart rate, blood pressure, and respiratory rate of less than 15% in response to the skin incision; and increases in the intraoperative heart rate, blood pressure, and respiratory rate of less than 25%.

The operation will be performed at least 15 min after the nerve block. During the operation, anesthesia will be maintained with 60% nitrous oxide, 40% oxygen, and 2% end-tidal sevoflurane. If the increases in heart rate, noninvasive blood pressure, and respiratory rate are greater than 15% from the baseline the values during the operation, the concentration of sevoflurane will be increased by 0.5%. Conversely, if the reductions in heart rate, noninvasive blood pressure, and respiratory rate are greater than 15% of the baseline values during the operation, the concentration of sevoflurane will be reduced by 0.5% [23]. If additional fentanyl is required when the heart rate, blood pressure, and respiratory rate increase by greater than 25% intraoperatively, the patient will be recorded as a failure or an inadequate lumbar plexus block and also be followed up. Immediate complications will be recorded and treated.

### Control group

Children assigned to the control group will receive a bolus injection of 1 μg/kg fentanyl intravenously before skin incision. During the operation, anesthesia will be maintained with 60% nitrous oxide, 40% oxygen, and 2% end-tidal sevoflurane. If the increases in the heart rate, noninvasive blood pressure, and respiratory rate are greater than 15% of the baseline values during the operation, then the concentration of sevoflurane will be increased by 0.5%. Conversely, if the heart rate, noninvasive blood pressure, and respiratory rate are reduced by greater than 15% of the baseline values during the operation, then the concentration of sevoflurane will be decreased by 0.5%. Furthermore, if the heart rate, blood pressure, and respiratory rate increase by greater than 25% of the baseline values during the surgery, then children will receive an additional bolus of 1 μg/kg fentanyl.

### Intraoperative anesthetic management and postoperative assessment

If the heart rate is reduced to less than 50 beats per min during the operation, then 10 μg/kg atropine will be used. If the blood pressure is reduced by greater than 25% of the baseline value, then 0.1 mg/kg ephedrine will be administered. At the end of surgery, sevoflurane will be discontinued, and the fresh gas flow will be increased to 6 l/min.

After completion of the operation, children will be transferred to the PACU and observed until the end of the study period. ECG results, noninvasive blood pressure, and pulse oximetry will also be measured every 5 min. After recovery of adequate of spontaneous breathing, purposeful movement, and eye opening, the endotracheal tube will be removed.

EA will be assessed using the PAED scale (Table 1) [24] at 0, 5, 10, 20, and 30 min after emergence from anesthesia. The PAED score will be documented by the same well-trained doctor, who will be blinded to the allocation group. The PAED score consists of five psychometric items: eye contact with the caregiver, purposeful action, awareness of surroundings, restlessness, and inconsolability. Each item is scored on a 5-point scale (0–4). The maximum score is 20. EA is defined as a PAED score greater than 10 points [25]. Patients with severe EA (PAED score ≥ 13) [24] will be treated with a bolus of 0.5 μg/kg fentanyl. The incidence of EA, frequency of severe EA, and duration of EA will be documented.

**Table 1:** Pediatric Anesthesia Emergence Delirium (PAED) scale

1. The child makes eye contact with the caregiver	
2. The child’s actions are purposeful	
3. The child is aware of his/her surroundings	
4. The child is restless	
5. The child is inconsolable	

Items 1, 2, and 3 are reverse scored as follows: 4 = not at all, 3 = just a little, 2 = quite a bit, 1 = very much, 0 = extremely

Items 4 and 5 are scored as follows: 0 = not at all, 1 = just a little, 2 = quite a bit, 3 = very much, 4 = extremely

Scores for each item are summed to obtain a total PAED score between 0 and 20

Postoperative pain will be evaluated using the CHEOPS by a blinded observer at 0, 5, 10, 20, and 30 min after emergence from anesthesia in the PACU. The CHEOPS is a pain-scoring method applied for children aged 1–7 years. The scale was established by McGrath et al. [26]. The CHEOPS consists of six items: crying, facial expression, verbalization, torso position, touching of the affected area, and leg movement. The maximum score is 13, and the minimum score is 4 points. Children with CHEOPS score ≥ 4 will be treated with a bolus of 0.5 μg/kg fentanyl.

Hemodynamic changes and complications will be recorded in the PACU and compared between the two groups.

### Data collection and management

Clinical characteristics include age, gender, weight, body mass index (BMI), ASA grade, duration of surgery, and duration of anesthesia (from induction of anesthesia to the discontinuation of sevoflurane).

Intraoperative data collection will include the following parameters: heart rate, noninvasive blood pressure, respiratory rate, dose of sevoflurane, and collective fentanyl dose.

Postoperative data collection will include the PAED score at every time point, incidence of EA, frequency of severe EA, duration of EA, time of emergence (from discontinuation of sevoflurane to the first response to a simple verbal command), extubation time (from the end of anesthesia to extubation), CHEOPS score at every time point, the incidence of complications and adverse events, and the dose of fentanyl in PACU.

### Sample size estimation

The sample size was determined by a power analysis based on the incidence of EA in our previous pilot study. The incidence of EA of the control group in our pilot study was approximately 42%. We expect that the intervention group will exhibit a 15% reduced incidence of EA. Based on these assumptions, we found that 45 patients per group will provide a power of 80% for detecting differences in EA incidence at a significance level of 0.05. Considering a possible dropout rate of 10%, 50 patients will be included per group.

### Statistical analysis

Patient demographics will be compared between the two groups to ensure that the data are balanced.

Intention-to-treat analysis will be used to analyze data including failed block. A per-protocol set will then be used to analyze data excluding the data for failed block. Continuous variables with a normal distribution will be reported as the means with the SD, which will be analyzed by an independent-samples *t* test. Data for the PAED and CHEOPS will be analyzed by either one-way repeated-measures ANOVA or Friedman’s repeated ANOVA on ranks according to the test for normality and homogeneity of variance. Categorical variables, such as the incidence of EA, will be reported as numbers and percentages, and analyzed by the chi-square test or Fisher’s exact test as appropriate. The reduction in pain, the decreased sevoflurane dose, and the dose of fentanyl in the PACU, regarded as covariates for the incidence of EA, will be assessed using logistic regression.

Statistical tests will be conducted using SPSS 12.0 (SPSS Inc., Chicago, IL, USA), and *P* < 0.05 will be considered statistically significant.

## Discussion

EA is a frequent problem in children receiving sevoflurane or desflurane anesthesia. Although some researchers have studied the effect of nerve block on EA, the results are uncertain. This trial seeks to determine whether ultrasound-guided lumbar plexus block administered to children undergoing elective hip surgery will result in a significantly reduced incidence of EA. When studying the effect of nerve block on EA, the type of operation performed should be considered because the site of the surgical procedure is significantly related to the frequency of EA [23]. Moreover, Ohashi et al. [23] reported that patients undergoing minimally invasive surgery may not experience sufficient pain for researchers to evaluate the effect of nerve block on EA. We chose orthopedic surgery because the rate of occurrence of EA is the third highest in this field [23], and hip surgery in children is not only a common pediatric procedure but is also associated with intense pain. Thus, we will not underestimate the effect of ultrasound-guided lumbar plexus block on EA. Ultrasound examination has revolutionized pediatric anesthesia, and ultrasound-guided nerve block has become more popular in pediatric orthopedic surgery in recent years given the increased safety and efficacy of the block. Previous trials have studied several superficial nerves. To the best of our knowledge, no study has assessed the effect of lumbar plexus block, which is a relatively deep procedure, on EA.

The results of our study will confirm the effect of lumbar plexus block on EA in pediatric hip surgery and provide clinical evidence for the prevention of EA. If our hypothesis is verified, then ultrasound-guided lumbar plexus block could be recommended as a safe prevention method for EA.

There are some limitations in our trial. First, this trial will be conducted in only one center, which may impact enrollment. In addition, the conclusions of our trial may not be applicable to other centers. However, the use of randomization and computer-generated allocation in our trial can reduce the risk of selection bias. In our trial, assessment investigators and data collectors will remain blinded to allocations, which reduces the treatment effect estimate bias. Moreover, standardization of intervention, including ultrasound-guided lumbar plexus block, intraoperative anesthetic management, and postoperative and outcome measurements, will be performed, which can avoid performance bias. In addition, a correct evaluation sample size was obtained according to our pilot study. Another limitation is that our trial assesses EA for only a short time after the operation, and the observation time is relatively short. The long-term prognosis of EA should be assessed in further studies.

### Trial status

This trial was initiated in May 2017 and is currently recruiting patients.

## Additional file

Additional file 1: SPIRIT checklist. (DOC 123 kb)

## Abbreviations

ASA: American Society of Anesthesiologists
CHEOPS: Children’s Hospital of Eastern Ontario Pain Scale
EA: Emergence agitation
ECG: Electrocardiography
PACU: Postanesthesia care unit
PAED: Pediatric Anesthesia Emergence Delirium
SpO_2_: Pulse oximetry
